# The After-Effects of Theta Burst Stimulation Over the Cortex of the Suprahyoid Muscle on Regional Homogeneity in Healthy Subjects

**DOI:** 10.3389/fnbeh.2019.00035

**Published:** 2019-03-01

**Authors:** Xiuhang Ruan, Guoqin Zhang, Guangqing Xu, Cuihua Gao, Lingling Liu, Yanli Liu, Lisheng Jiang, Sijing Zhang, Xin Chen, Xinqing Jiang, Yue Lan, Xinhua Wei

**Affiliations:** ^1^Department of Radiology, Guangzhou First People’s Hospital, Guangzhou Medical University, Guangzhou, China; ^2^Department of Rehabilitation Medicine, Beijing Tiantan Hospital, Capital Medical University, Beijing, China; ^3^China National Clinical Research Center for Neurological Diseases, Beijing, China; ^4^Department of Rehabilitation Medicine, Guangzhou First People’s Hospital, Guangzhou Medical University, Guangzhou, China; ^5^The Second Affiliated Hospital, South China University of Technology, Guangzhou, China

**Keywords:** theta burst stimulation, functional magnetic resonance, resting state, regional homogeneity, swallowing

## Abstract

Theta burst stimulation (TBS) is a powerful variant of repetitive transcranial magnetic stimulation (rTMS), making it potentially useful for the treatment of swallowing disorders. However, how dose TBS modulate human swallowing cortical excitability remains unclear. Here, we aim to measure the after-effects of spontaneous brain activity at resting-state using the regional homogeneity (ReHo) approach in healthy subjects who underwent different TBS protocols over the suprahyoid muscle cortex. Sixty healthy subjects (23.45 ± 2.73 years, 30 males) were randomized into three groups which completed different TBS protocols. The TMS coil was applied over the cortex of the suprahyoid muscles. Data of resting-state functional MRI (Rs-fMRI) of the subjects were acquired before and after TBS. The ReHo was compared across sessions [continuous TBS (cTBS), intermittent TBS (iTBS) and cTBS/iTBS] and runs (pre/post TBS). In the comparison between pre- and post-TBS, increased ReHo was observed in the right lingual gyrus and right precuneus and decreased ReHo in the left cingulate gyrus in the cTBS group. In the iTBS group, increased ReHo values were seen in the pre-/postcentral gyrus and cuneus, and decreased ReHo was observed in the left cerebellum, brainstem, bilateral temporal gyrus, insula and left inferior frontal gyrus. In the cTBS/iTBS group, increased ReHo was found in the precuneus and decreased ReHo in the right cerebellum posterior lobe, left anterior cerebellum lobe, and right inferior frontal gyrus. In the post-TBS inter-groups comparison, increased ReHo was seen in right middle occipital gyrus and decreased ReHo in right middle frontal gyrus and right postcentral gyrus (cTBS vs. cTBS/iTBS). Increased ReHo was shown in left inferior parietal lobule and left middle frontal gyrus (cTBS vs. iTBS). Increased ReHo was shown in right medial superior frontal gyrus and decreased ReHo in right cuneus (cTBS/iTBS vs. iTBS). Our findings indicate cTBS had no significant influence on ReHo in the primary sensorimotor cortex, iTBS facilitates an increased ReHo in the bilateral sensorimotor cortex and a decreased ReHo in multiple subcortical areas, and no reverse effect exhibits when iTBS followed the contralateral cTBS over the suprahyoid motor cortex. The results provide a novel insight into the neural mechanisms of TBS on swallowing cortex.

## Introduction

Swallowing is a complex activity involving a widely distributed neuronal network, and a body of evidence indicates that cortical and subcortical areas play a crucial role in swallowing control (Hamdy et al., 1999b; Humbert and Robbins, 2007; Martin et al., 2007). Dysphagia is a major complication that occurs in 37%–78% of stroke patients (Martino et al., 2005). Post-stroke dysphagia may lead to severe complications including aspiration pneumonia, dehydration and malnutrition, which are associated with an increased risk of mortality (Martino et al., 2005; Cabib et al., 2016). The usual clinical practice to manage post-stroke dysphagia is to provide nutritional support *via* alternative feeding methods and behavioral adaptations (e.g., modifying food consistencies, compensatory maneuvers); however, the efficacy of these methods is controversial and needs high-quality evidence to verify it (Bath et al., 2000).

Against this background, development of an effective intervention that improve swallowing function through promoting functional recovery with concept of neuroplasticity in the early course of stroke will be helpful in restoring swallowing functions of post-stoke dysphagia (Pisegna et al., 2016). Transcranial magnetic stimulation (TMS) is a noninvasive brain stimulation modality in which a magnetic field generates electrical current in the brain to polarize neurons in the underlying cortex (Sandrini et al., 2011), and has exhibited substantial potential in dysphagia treatment (Pisegna et al., 2016). The potential neural mechanism may be connected with rebalance of interhemispheric interactions because the evidence indicates that and interhemispheric imbalance hinders motor function recovery in dysphagic patients (Khedr et al., 2008). Moreover, there exists a newly developed TMS protocol called theta burst stimulation (TBS), which has robust and long-lasting effects, with a low intensity and high frequency benefit to the recovery of motor and linguistic function in chronic stroke patients (Talelli et al., 2007). Interestingly, different patterns of delivery of TBS produce opposite after-effects on synaptic efficiency of the stimulated cortex. The paradigm termed intermittent TBS (iTBS) induces long-term potentiation (LTP) effect and improves cortical excitability (Larson et al., 1986). By contrast, continuous TBS (cTBS) involves uninterrupted TBS trains and leads long-term depression (LTD) and has shown to decrease cortical excitability (Huang et al., 2005). However, there remain a number of unanswered questions related to the neural mechanisms of TBS and the entity of excitatory and inhibitory stimulation, and little is known about either the potential therapeutic mechanisms for TBS treatment or the alteration in swallowing musculature anatomical representation in the human cerebral cortex. Only a few studies have attempted to explore the after-effects induced by TBS on cortical excitability by using motor-evoked potentials (MEPs) for recording, whereas the effects of cortical spontaneous brain activation have not been assessed (Mistry et al., 2007, 2012).

Recently, resting-state fMRI (Rs-fMRI) has been applied to detect spontaneous brain activity for exploring the neural basis of various brain disorders (Huang et al., 2010; Zhang et al., 2011; Meng et al., 2014). Functional connectivity is the most commonly used approach, which examines the temporal relationships between fluctuations observed in spatially distinct brain regions (Biswal et al., 1995; Fox and Greicius, 2010; Lee et al., 2012). However, functional connectivity approach provides little information about local features of spontaneous brain activity observed in individual regions (Liu et al., 2010). As a novel analysis method, regional homogeneity (ReHo) is a local measurement of functional connectivity, measures the similarity or synchronization of the time series of a given voxel to those of its nearest neighbors in a voxel-wise manner (Zang et al., 2004). The ReHo approach has been applied to explore local abnormalities in brain disorders (Wu et al., 2009; Guo et al., 2011; Liao et al., 2012). Our group explored the alterations of amplitude of low frequency fluctuation (ALFF) with different protocols of TBS on swallowing cortex in a previous pilot study (Ruan et al., 2017). However, ReHo and ALFF are quite different methods. ReHo approach focus on the similarities of intraregional time series, and ALFF measures the amplitude of regional activity (Yuan et al., 2013). More recently, there have been an increasing number of Rs-fMRI studies that have started to investigate the neural mechanisms of noninvasive brain stimulation (Valchev et al., 2015; Hartwright et al., 2016; Mancini et al., 2017). Nevertheless, few studies have investigated cortical excitability or the after-effects after implement of different TBS protocols on the motor cortex of deglutition during the resting state (Ruan et al., 2017). How does the TBS take effect on spontaneous brain activity and which brain area demonstrates alteration in neuronal activity remains unclear, whereas this knowledge could be useful for optimizing therapeutic applications of TBS, particularly in poststroke dysphagia.

Our group investigated TBS effects on cortex excitability using MEPs in a previous study (Lin et al., 2017), which indicated that TBS could effectively regulated suprahyoid motor cortex excitability. The MEP is powerful in verifying the final after-effect of TBS on the swallowing motor cortex. However, the underlying neural mechanism related to TBS on the swallowing motor cortex needs further research. This study aimed to measure the alterations in ReHo induced by different patterns of stimulation, including single used iTBS and cTBS, and a combination of iTBS and cTBS. The target site for TBS stimulation was the motor cortex of the suprahyoid muscles, which play an important role in movement of the hyoid-throat complex (Nam et al., 2013). As inhibitory pre-conditioning protocol of TMS, such as cTBS leads temporarily disrupts normal cortical excitability and can be used as a “virtual lesion” to assess the efficacy of neurostimulation techniques before progressing to patient trials (Vasant et al., 2014). Therefore, another purpose was that we used the cTBS to create a “virtual lesion” in the ipsilateral swallowing motor cortex for exploring the after-effect of iTBS which was placed on the contralateral brain area. Furthermore, given the evidence that excitatory TMS over contralesional pharyngeal motor cortex was benefit to the recovery of in post-stroke patients with dysphagia (Park et al., 2013; Michou et al., 2016). We were interested in whether iTBS could reverse the inhibitory effect induced by cTBS on the contralateral suprahyoid motor cortex. According to our previous findings (Lin et al., 2017; Ruan et al., 2017), we hypothesized that iTBS, cTBS will induce distinct alternations of ReHo in multiple brain areas. In addition, we expect iTBS will reverse the inhibitory effect induced by cTBS on the contralateral suprahyoid motor cortex.

## Materials and Methods

### Participants

We studied 60 healthy subjects (mean age: 23.5 ± 4.4 years, 30 females). All were right handed according to the Edinburgh Handedness Inventory and had normal neurological and general medical examinations. Participants were free of any neurological or psychiatric disease, swallowing disorders, drug or alcohol abuse, and exposure to neuropsychiatric medications. The subjects were randomized into cTBS group (*n* = 20), iTBS group (*n* = 20) or cTBS/iTBS group (*n* = 20). Written informed consent was obtained from all subjects prior to participation, and the study was performed in accordance with the World Medical Association Declaration of Helsinki and approved by the Institutional Review Board for the Protection of Human Subjects at Guangzhou First People’s Hospital.

### Transcranial Magnetic Stimulation

#### Electromyography (EMG) Recording

The electromyography (EMG) responses of suprahyoid muscle were detected by two pairs of bipolar silver-silver chloride electrodes (Yiruide, Wuhan, China), which is placed on the left and right sides of the suprahyoid muscle groups projection area. A pair of electrodes was attached to the abductor muscle of the thumb for measuring the threshold of motion. All electrodes were connected to an EMG recording system (Yiruide, Wuhan, China).

#### Determination of Motion Threshold

Focal TBS was delivered through a Magstim super rapid stimulator (Yiruide medical equipment Co., Wuhan, China) connected to a figure-of-eight coil 70 mm in diameter. Neuronavigation (Softaxi Optic, Canada, NDI) was used to position the stimulator at the motor cortex of the suprahyoid muscles. The subject was seated on a chair with armrest, and the coil was placed toward the target hemisphere according to navigation system and was kept in contact with the scalp closely. Single pulse stimulation is triggered from 60% of maximum output intensity, and the stimulation intensity is gradually increased until a significant left abduction activity is induced, and then the stimulation intensity is maintained, each time with a slight distance of 0.5–1.0 cm. Moving the coil for five consecutive stimulations, the position of the maximal MEP amplitude and the shortest latency period is considered to be the maximum motion stimulation zone of the left thumb abductor muscle. Fix the coil and gradually reduce the stimulation intensity until at least five times of 10 consecutive stimulations can induce MEP of ≥50 μV in the left thumb abductor muscle. This stimulation intensity is the resting motion threshold of the subject (Rest Motor Threshold, RMT). Move the coil to the anterolateral side and give single pulse stimulation with 70% output intensity. Move the coil slightly at a distance of 0.5–1.0 cm each time. The position of the MEP amplitude induced by the five consecutive stimulations, which is regarded as the best stimulation point of the suprahyoid muscle groups. The position of the MEP optimal stimulation point on the left and right suprahyoid muscle groups is preserved by the nerve positioning navigation system to ensure that the subsequent stimulation sites are consistent.

#### TBS Stimulation Protocol

Briefly, we verified the stimulating protocol efficacy by measuring MEPs. Repetitive TMS (rTMS) was delivered using the TBS paradigm which consisted of bursts that contained three pulses at 50 Hz repeated at 5 Hz. During the iTBS, each burst of burst stimuli consisted of three consecutive pulses, 2 s stimulation, 8 s intermittent, and repeated 20 times for approximately 190 s (600 pulses). For the cTBS paradigm, each burst of burst stimuli consisted of three consecutive pulses with 200 bursts of intermittent stimulation for a total of 600 pulses with a sustained stimulation time of 40.04 s (Bertini et al., 2010).

#### Experimental Protocols

The three groups were subjected to different protocols in the current study; in group 1 and group 2, cTBS and iTBS were positioned on the left cortex of the suprahyoid muscles. It was reported that the majority of subjects lateralized to the left hemisphere for the pharynx, right suprahyoid and left suprahyoid muscle sites. Therefore, in the present study, we chose the left hemisphere as the target. In group 3, after the cTBS placed on the left cortex of suprahyoid muscles, the iTBS was immediately delivered on the right suprahyoid muscle cortex. In this protocol, the cTBS was used to create a “virtual lesion” in the left side swallowing motor cortex and the iTBS was placed on the right side to explore whether iTBS could reverse the inhibitory effect of cTBS in the contralateral hemisphere.

#### Imaging Data Acquisition

The baseline and post-TBS MR studies were conducted within an interval of 2 h in the same day. Participants were scanned on a Siemens Verio 3.0 T scanner (Siemens, Erlangen, Germany). A high resolution T1-weighted images were obtained in an axial orientation [repetition time (TR) = 2,530 ms, echo time (TE) = 2.93 ms, flip angle (FA) = 7°, field of view (FOV) = 256 mm × 256 mm, slice thickness = 1.0 mm, no slice gap]. Functional images were obtained by using an echo-planar imaging sequence (33 axial slices, TR = 2,000 ms, TE = 21 ms, FA = 90°, FOV = 240 mm × 240 mm, matrix = 64 × 64, slice thickness = 4.0 mm, voxel size = 3.75 mm ×3.75 mm × 4.0 mm).

#### Image Preprocessing

Image processing was performed using the DPARSF software package^1^ (Chao-Gan and Yu-Feng, 2010). For each participant, the first 10 images of each dataset were discarded to allow for magnetization equilibrium and for the participants to adjust to the environment. All subjects had less than 2 mm maximum displacement in x, y, or z and 2° of angular motion during the whole fMRI scan. Then, the images were normalized to the standard SPM8 echo-planar imaging template, resampled with voxel size of 3 mm × 3 mm × 3 mm. The white matter signal, cerebrospinal fluid signal and Friston 24 motion parameters were removed by regression. Linear trend subtraction and temporal filtering (0.01–0.08 Hz) were carried out on the time series of each voxel to reduce the effects of low-frequency drifts and high-frequency respiratory and cardiac noise (Biswal et al., 1995).

#### ReHo Analysis

The REST software package^2^ was used to calculate the ReHo values and generate the ReHo maps. The details of ReHo analysis were described in previous studies (Zang et al., 2004; Wu et al., 2009). Briefly, the ReHo maps were produced by calculating the Kendall coefficient of concordance (KCC) which measures the similarity between the time series of a given voxel and those of its 26 nearest neighbors (Zang et al., 2004). Then, each individual ReHo map was divided by its own global mean KCC value within the brain mask for standardization (Wang et al., 2014). Finally, the standardized ReHo images were smoothed using a Gaussian kernel of full-width athalf-maximum 4.0 mm.

#### Statistical Analysis

Demographics, including age and gender of all subjects, were analyzed using SPSS, version 16.0 (SPSS Inc., Chicago, IL, USA). The continuous variables of the three groups were compared using one-way analysis of variance (ANOVA), and the chi-square test was used to analyze the categorical data.

The ReHo comparisons were used the DPABI software package^3^ (Yan et al., 2016). We performed an inter-groups comparison of the ReHo maps of subjects between the post-TBS and baseline using paired *t*-tests in the three groups. We examined the normalized ReHo maps in a voxel-by-voxel manner with regression of age, gender and gray matter volume. The results were thresholded with *p* < 0.05 with a combined individual cluster size >85 voxels corrected using Monte Carlo simulations (see AlphaSim program in AFNI^4^ to minimize type I errors. The ANOVA test was performed for comparing the main effects of ReHo maps among the three groups with post-TBS using DPABI software package (Yan et al., 2016), and *post hoc* analysis was then conducted to investigate the differences of ReHo maps between paired groups (*p* < 0.05 with AlphaSim correction and a cluster size >85 voxels).

## Results

Demographic information is presented in Table 1. No significant difference in age and gender was observed among the three groups (*p* > 0.05).

**Table 1:** Demographic information of study subjects.

Characteristic	cTBS group (*n* = 20)	iTBS group (*n* = 20)	cTBS/iTBS group (*n* = 20)	*p* value
Gender: male/female	10/10	10/10	10/10	1.00^a^
Age, years: mean (SD)	23.60 ± 2.23	22.95 ± 2.67	23.80 ± 3.27	0.60^b^

*Abbreviations: cTBS, continuous theta-burst stimulation; iTBS, intermittent TBS; SD, standard deviation; ^a^indicate the *p* value for the Chi-Square test; ^b^*p* value for the analysis of variance (ANOVA) *F*-test*.

**Figure 1:**
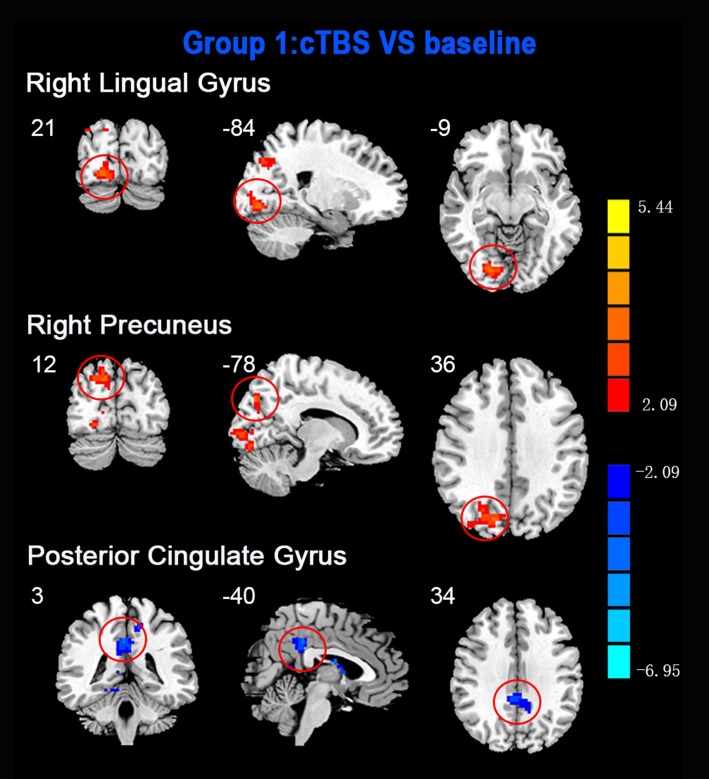
Statistic maps showing regional homogeneity (ReHo) differences between continuous theta burst stimulation (cTBS) and baseline. Regions with red color represent significantly increased ReHo values in the TBS compared with the baseline, and blue indicate the opposite (*p* < 0.05, corrected). The details are described in Table 2. Color bar indicates the *t* score.

**Figure 2:**
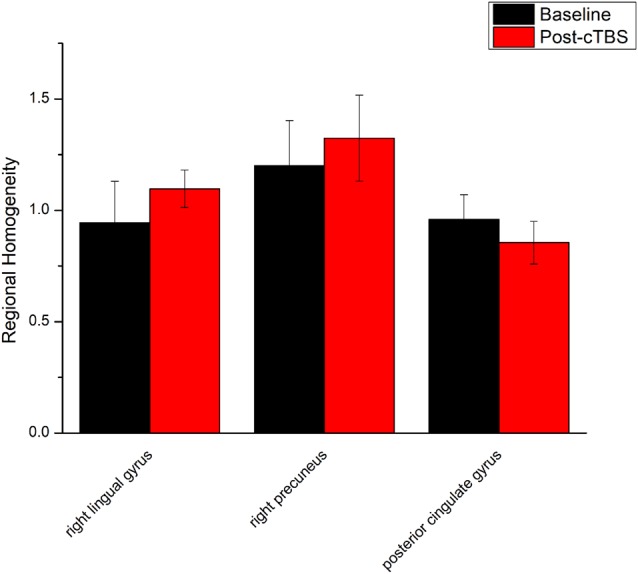
Comparison of ReHo values between cTBS and baseline. There were significant difference in ReHo values between cTBS and baseline in three brain regions.

**Figure 3:**
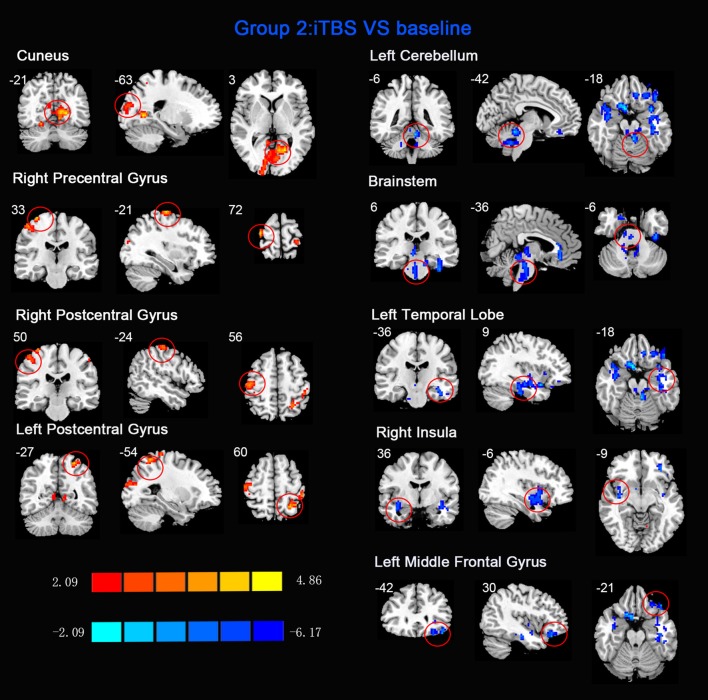
Statistic maps of ReHo between intermittent TBS (iTBS) and baseline. The illustration of Figure 3 is the same as that in Figure 1. The details are displayed in Table 2.

**Figure 4:**
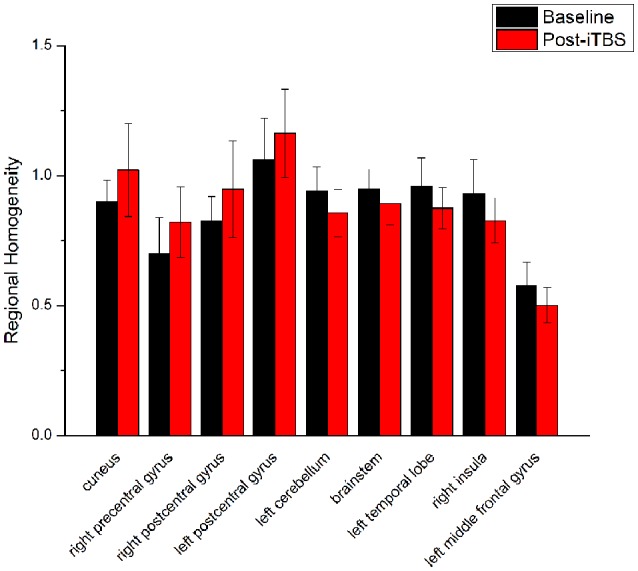
Comparison of ReHo values between iTBS and baseline. Significant differences in ReHo values between iTBS and baseline were observed in nine brain areas.

**Figure 5:**
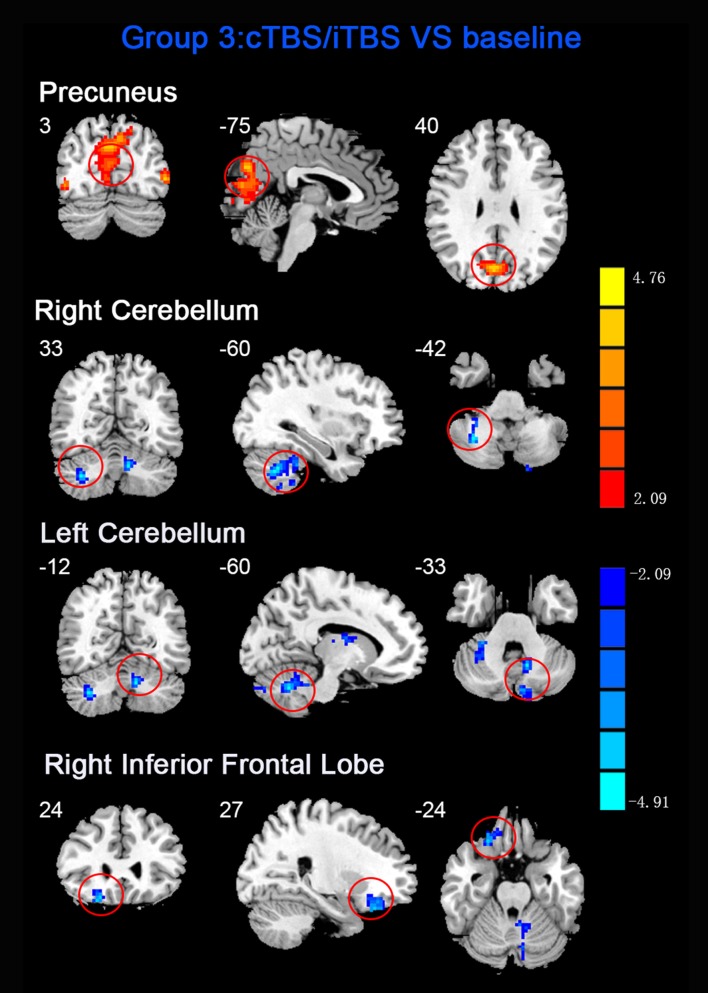
Statistic maps of ReHo between cTBS/iTBS and baseline. The illustration of Figure 5 is the same as that in Figure 1. The details of brain areas are presented in Table 2.

**Figure 6:**
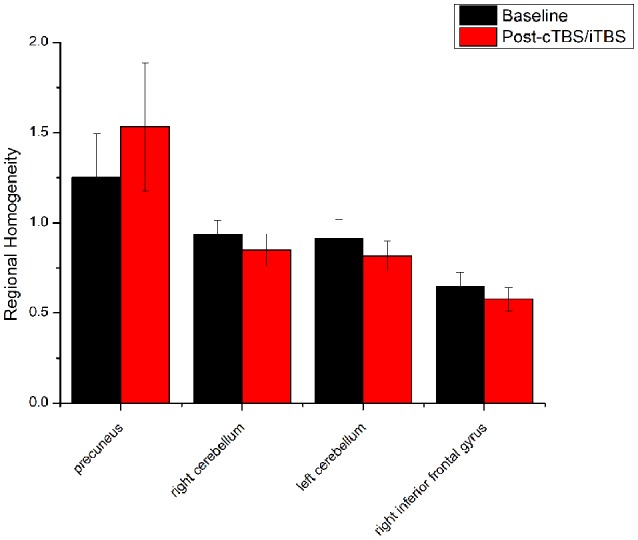
Comparison of ReHo values between cTBS/iTBS and baseline applied on the cortex of suprahyoid muscles. Four brain regions demonstrated significant difference in ReHo values between cTBS/iTBS and baseline.

ReHo comparisons were made between post-TBS and pre-TBS conditions in the three groups. In group 1, compared to the condition of pre-cTBS, post-cTBS showed increased ReHo in the right lingual gyrus (BA 18) and right precuneus (BA 19) and decreased ReHo in the posterior cingulate gyrus (BA 23; Figures 1, 2, Table 2). In group 2, compared to the baseline, post-iTBS induced an increase in ReHo in the bilateral precentral gyrus (BA 6), left postcentral gyrus (BA 7), and cuneus (BA 19), and decreased ReHo was shown in the left cerebellum, brainstem, left temporal gyrus (BA 38), right insula (BA 48), and left middle frontal gyrus (BA 38; Figures 3, 4, Table 2). In group 3, compared to the baseline, post-combined cTBS/iTBS exhibited increased ReHo in the precuneus (BA 7) and decreased ReHo in the right cerebellum posterior lobe, left cerebellum anterior lobe, and right inferior frontal gyrus (BA 11; Figures 5, 6, Table 2).

**Table 2:** Brain regions with alteration of ReHo after different TBS protocols over the motor cortex of suprahyoid muscles.

Brain region	BA	Cluster size (voxels)	Peak MNI coordinates (mm)			*T* value
			*X*	*Y*	*Z*	
**cTBS vs. baseline**						
Right lingual gyrus	18	86	21	−84	−9	3.4455
Right precuneus	19	90	12	−78	36	3.5117
Posterior cingulate	23	103	3	−40	34	−5.1444
**iTBS vs. baseline**						
Cuneus	19	558	−21	−63	3	4.5645
Right precentral gyrus	6	62	33	−21	72	4.0946
Right postcentral gyrus	3	63	50	−24	56	4.0946
Left postcentral gyrus	7	61	−27	−54	60	4.1244
Left cerebellum	N/A	84	−6	−42	−18	−5.1949
Brainstem	N/A	149	6	−36	−6	−3.7978
Left temporal lobe	38	155	−36	9	−18	−5.0445
Right insula	48	72	36	−6	−9	−3.8814
Left middle frontal gyrus	38	125	−42	30	−21	−4.5231
**cTBS/iTBS vs. baseline**						
Precuneus	7	235	3	−75	40	4.2472
Right cerebellum	N/A	124	33	−60	−42	−4.7972
Left cerebellum	N/A	155	−12	−60	−33	−4.6053
Right inferior frontal lobe	11	91	24	27	−24	−4.3816

*Abbreviations: ReHo, regional homogeneity; cTBS, continuous theta-burst stimulation; iTBS, intermittent TBS; BA, Brodman’s area; MNI, Montreal Neurological Institute; N/A, not available*.

**Figure 7:**
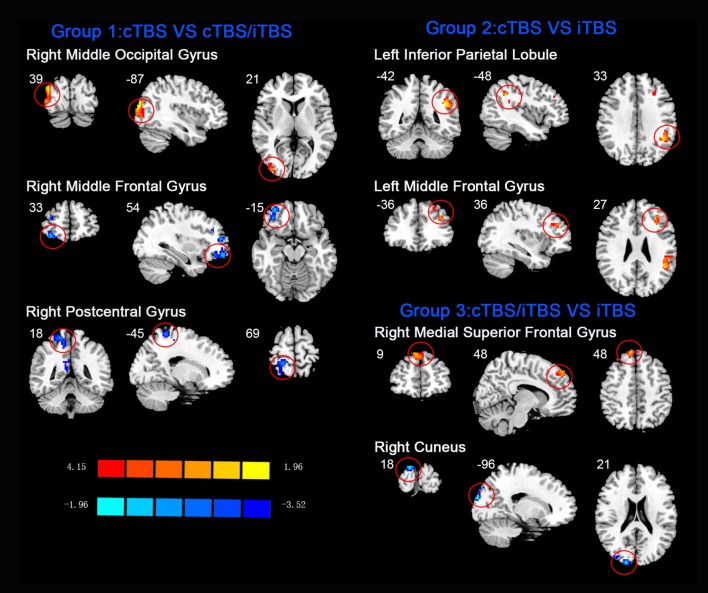
Comparison of ReHo values among three different protocols of TBS applied on the cortex of suprahyoid muscles.

In the comparison between pre- and post- TBS, increased ReHo was seen in right middle occipital gyrus (BA 19) and decreased ReHo in right middle frontal gyrus (BA 11) and right postcentral gyrus (BA 5; post-cTBS vs. post-cTBS/iTBS). Increased ReHo was shown in left inferior parietal lobule (BA 39) and left middle frontal gyrus (BA 46; post-cTBS vs. post-iTBS). Increased ReHo was shown in right medial superior frontal gyrus (BA 9) and decreased ReHo in right cuneus (BA 18; post-cTBS/iTBS vs. post-iTBS; Figure 7, Table 3).

**Table 3:** ReHo comparison among different protocols of TBS on the cortex of suprahyoid muscles.

Brain region	BA	Cluster size (voxels)	Peak MNI coordinates (mm)			*T* value
			*X*	*Y*	*Z*	
**cTBS vs. cTBS/iTBS**						
Right middle occipital gyrus	19	87	39	−87	21	4.0336
Right middle frontal gyrus	11	135	33	54	−15	−3.625
Right postcentral gyrus	5	61	18	−45	69	−3.0683
**cTBS vs. iTBS**						
Left inferior parietal lobule	39	205	−42	−48	33	4.0544
Left middle frontal gyrus	46	74	−36	36	27	3.2247
**cTBS/iTBS vs. iTBS**						
Right medial superior frontal gyrus	9	72	9	48	48	3.5708
Right cuneus	18	49	18	−96	21	−5.5187

*Abbreviations: ReHo, regional homogeneity; cTBS, continuous theta-burst stimulation; iTBS, intermittent TBS; BA, Brodman’s area; MNI, Montreal Neurological Institute*.

## Discussion

### The After-Effect on ReHo Between Different Protocols of TBS and Baseline

As a pattern of inhibitory stimulation, we expected that cTBS would produce suppressive after-effects of local cortical excitability in the target position. However, similar to a previous study (Mistry et al., 2007), after cTBS application over the left motor cortex of the suprahyoid muscles, we did not observe altered ReHo in the stimulation position or the contralateral homologous area. It was speculated that the circuitry between the I1 input and the corticospinal neurons may be less susceptible to the type of inhibition produced by TBS over the pharyngeal motor cortex (Mistry et al., 2007). In addition, we found increased ReHo in the right lingual gyrus and right precuneus belonging to the right occipitoparietal regions, which was reported in previous fMRI studies (Martin et al., 2007). Specifically, the lingual gyrus was activated in voluntary saliva swallowing and probably played a role in deglutition (Martin et al., 2007), and the precuneus is part of the posterior association area of the brain that integrates information emanating from more than one sensory modality (Van Hoesen, 1993). Hence, the occipitoparietal regions are thought to be a general hub which plays a role in processing and integrating sensory input with motor output as well as the association of visual and auditory cues to sensory/motor response (Kern et al., 2001). Furthermore, we observed decreased ReHo in the posterior cingulate gyrus, which is believed to be an association area that has reciprocal connections with the thalamus and plays an active role in integrating sensory information (Yukie, 1995). Activation of this area, along with other sensory areas (precuneus, thalamus) in swallowing, could indicate they play a role in receiving and processing sensory information from the oropharyngeal areas and modulating motor activities through connections with the primary motor cortex and insula (Hamdy et al., 1999b). Together, we speculated that cTBS might have no significant influence on local cortical excitability on the primary motor/sensor areas, but induce after-effects on the posterior association area, which is involved in processing and integrating sensory input with motor output to adjust the activity of deglutition.

When we applied iTBS on the left cortical area of the suprahyoid muscle, as expected, we observed alterations in ReHo in the bilateral primary motor/sensory cortex, which was consistently activated across healthy subjects during swallow-task fMRI studies (Humbert and Robbins, 2007; Martin et al., 2007). Given that swallowing musculatures are bilaterally and often asymmetrically represented within the brain (Hamdy et al., 1996), we speculated that the transcallosal inhibition is of less importance in a bilaterally represented hemispheric control system than in a unilaterally controlled system in swallowing (Mistry et al., 2012). Generally, the caudolateral motor cortex may be associated with the initiation of the swallowing sequence at the highest level (Ertekin and Aydogdu, 2003), and the primary somatosensory cortex may reflect various types of oropharyngeal sensory processing. This hypothesis underscores the importance of afferent information in the regulation of both voluntary and automatic swallowing (Sandrini et al., 2011). Additionally, compared to the baseline, we observed decreased ReHo in multiple subcortical and medial cortical regions, including the left cerebellum, brainstem, bilateral temporal gyrus and insula, and the left inferior frontal gyrus. A previous PET study indicated that the cerebellum plays an important role in the regulation of human swallowing (Hamdy et al., 1999b). In addition, other functional neuroimaging studies demonstrated that activation of the cerebellar hemispheres was induced by chewing (Onozuka et al., 2002), orofacial movements (Sörös et al., 2006), lip and tongue movements (Grodd et al., 2001), and whistling (Dresel et al., 2005). It is not surprising that the swallowing activity is mediated primarily by the brain stem (Torii et al., 2012). The interneurons or premotor neurons of deglutition are principally located in two regions of the brain stem in anatomy (Umezaki et al., 1998; Jean, 2001). Furthermore, the medullary network of deglutition can be activated by cortical commands (Ertekin, 2011). Similar to a previous study (Hamdy et al., 1999a), the temporal lobe and insula were consistently reported to be involved in swallowing (Hamdy et al., 1999b). A PET study suggested that the anteromedial temporal lobe is involved in human taste quality recognition (Small et al., 1997). The insular lobe is thought to involve sensorimotor integration, auditory and speech processing. Furthermore, neuroimaging studies indicate the frontal operculum/insula and the orbitofrontal are involved in taste tasks (Ertekin and Aydogdu, 2003). According to the above findings, we speculated that iTBS might influence both sides of the primary motor/sensory cortex, and the higher centers of the pre/post central gyrus might have negative feedback regulations of input and output with the brain stem and other subcortical areas (Mosier and Bereznaya, 2001).

The third protocol in the present study was combined use of cTBS and iTBS, i.e., cTBS was placed on the left motor area of the suprahyoid muscles followed by iTBS on the corresponding area of the right side. The purpose of this protocol was to verify our hypothesis that whether the contralateral iTBS could reverse the after-effects induced by cTBS. We applied the cTBS on the left motor areas of the suprahyoid muscles to create a virtual lesion which temporarily disrupts normal cortical excitability (Cugy et al., 2016; Michou et al., 2016). After that, the subsequent application of contralateral iTBS is thought to facilitate synaptic transmission within the available neuronal pool (Talelli et al., 2007). However, in contrast to our previous results identified by MEPs (Lin et al., 2017), we did not find significant alterations in ReHo in the bilateral pre-/postcentral gyrus when compared to the baseline. Instead, we found decreased ReHo in the cerebellum and inferior frontal gyrus, which were similar to the iTBS protocol we mentioned in above. Moreover, significantly increased ReHo was shown in the precuneus, which was similar to that showed in the cTBS protocol. We speculate that the iTBS has no significant reverse effect on the contralateral cTBS (virtual lesions), and we guess that the after-effects of both TBS between the suprahyoid motor cortices would eventually restore balance after the interhemispheric interactions. Another explanation for these finding is that we used the static ReHo method to analyze the Rs-fMRI data, in which we only use the mean information from the whole scanning time. Perhaps a dynamic analysis would be more helpful to identify the dynamic alteration in neural activity during the scanning time. Interesting, compare with our previous pilot study (Ruan et al., 2017), there is quite diverse in the alteration of brain areas between the usage with ReHo and ALFF for detecting the after-effect of different protocols of TBS. In fact, ReHo is quite different from ALFF method. ReHo analyses focus on the similarities of intra-regional time series, and ALFF measures the amplitude of regional activity (Yuan et al., 2013).

### Comparison of After-Effect of Different Protocols of TBS on ReHo Among Groups

Apart from analysis between pre- and post-TBS, the ReHo alterations induced by different protocols of post-TBS were explored. When cTBS compared with cTBS/iTBS protocol, decreased ReHo in right postcentral gyrus and middle frontal gyrus, and increased ReHo were seen in right middle occipital gyrus. We suppose that decreased ReHo in postcentral gyrus is due to the after-effect of iTBS targeted on the right swallowing motor cortex. As we mentioned above, Primary sensory area plays an important role in afferent information in automatic and voluntary swallowing regulation (Martin et al., 2001). The middle frontal gyrus is believed to be associated with planning of sequential movements, in particular, as occurs with swallowing (Tanji et al., 1996). Middle occipital gyrus is part of parieto-occipital regions which might play a role in reception and higher processing of sensation from the oropharyngeal and esophageal areas (Hamdy et al., 1999a). For the comparison between cTBS and iTBS protocols, we found increased ReHo was shown in left inferior parietal lobule and middle frontal gyrus. Inferior parietal lobule is considered a component of the brain swallowing network and has been viewed as a projection area of visceral stimulation (Babaei et al., 2013), and the middle frontal gyrus displayed activity after water or saliva swallowing (Sörös et al., 2009). Moreover, the prefrontal cortex was related to perception of body signals, attentional control, and higher order sensorimotor processing (Suntrup et al., 2014). In addition, Increased ReHo was shown in right medial superior frontal gyrus and decreased ReHo in right cuneus when compared cTBS/iTBS with iTBS protocols. Superior frontal gyrus have been shown to be involved in the complexity of the phases of swallowing, indicating that this area may play a role in motor planning of complex sequential movements (Haupage et al., 2010). As for cuneus, is a part of parietal-occipital regions, might play a role in reception and sensation in swallowing activity (Hamdy et al., 1999a). To summarize, when compared with the different protocols of TBS, alteration of ReHo was observed in multiple brain areas including frontal, parietal and occipital gyrus which involved in swallowing processing or other nonspecific functions.

There are some limitations in our study. First, considering that a previous study confirmed that sham TBS does not alter cortical excitability (Mistry et al., 2012), we did not design a sham stimulation to compare the after-effects of TBS. Second, given that we explored the TBS effects on suprahyoid motor cortex excitability by using suprahyoid MEPs (SMEPs) in our previous study (Lin et al., 2017), we did not record the SMEP after the TBS stimulation in the present study. Third, considering the tolerance and cooperation of the subjects during Rs-fMRI scanning, we did not measure the after-effects of TBS until the effects of TBS faded away. Fourth, the participants of this study were relatively young; however, swallowing disorders are more commonly seen in older patients. Thus, the recruitment of older subjects for further study is needed to verify the present results. Finally, our sample size was relatively small, and this might be one of the reasons for the underpowered results in the present study.

In conclusion, our findings indicate that the ReHo approach facilitates detecting the after-effects of TBS on the suprahyoid motor cortex. In our study, cTBS had no significant influence on ReHo in the primary sensorimotor cortex. However, iTBS facilitated an increased ReHo in the bilateral sensorimotor cortex and generated a decreased ReHo in multiple subcortical areas. Additionally, we could not find the reverse effect in ReHo as we expected when iTBS followed the contralateral cTBS over the suprahyoid motor cortex. Our findings provide novel evidence for detecting the alteration in spontaneous brain activation induced by different patterns of TBS on the swallowing motor cortex. These results have therapeutic potential as an adjunctive treatment for dysphagia when TBS is applied over the swallowing cortex.

## Author Contributions

XW contributed in the experimental design. XR and GZ contributed in writing of the manuscript. CG, YLi and XR were involved in literature review, data collection, and writing of the manuscript. LL and XC contributed to the analysis of MRI data. LJ, YLi and SZ were involved in the data collection. GX, XJ and YLa contributed in the experimental design, and in the writing process.

## Conflict of Interest Statement

The authors declare that the research was conducted in the absence of any commercial or financial relationships that could be construed as a potential conflict of interest.

## Footnotes

^1^http://rfmri.org/DPARSF

^2^http://www.restfmri.net/forum/

^3^http://rfmri.org/dpabi

^4^http://afni.nimh.nih.gov/pub/dist/doc/manual/AlphaSim.pdf
